# A Rare Case of Schwannoma at the Glans Penis: Comprehensive Clinical and Radiological Evaluation, Surgical Management, and Long-Term Follow-Up

**DOI:** 10.7759/cureus.103813

**Published:** 2026-02-17

**Authors:** Sachi Sankhala, Shailesh Raina

**Affiliations:** 1 Urology, Jaslok Hospital and Research Centre, Mumbai, IND

**Keywords:** contrast enhanced multiplanar mri, neurilemmomas, penis, schwannomas, ultrasonography

## Abstract

Schwannomas (also known as neurilemmomas) are benign peripheral nerve sheath tumors that arise from Schwann cells. Although they can occur throughout the body, penile involvement is exceedingly rare. Among these, schwannomas of the glans penis are exceptionally uncommon. Their nonspecific presentation often poses a diagnostic challenge, making accurate preoperative identification difficult.

We report the case of a sexually active 33-year-old man who presented with a painless, slowly enlarging mass on the dorsal aspect of the glans penis. Physical examination revealed a solitary, well-circumscribed, soft to firm, non-tender nodule measuring approximately 2 × 2 cm, without overlying skin changes or regional lymphadenopathy. Ultrasonography demonstrated a hypoechoic lesion with internal septations and vascularity. Contrast-enhanced multiplanar MRI of the penis showed a 2.1 × 1.7 × 1.7 cm well-circumscribed, avidly enhancing soft-tissue mass with internal cystic/necrotic areas, abutting but not invading the urethra or terminal penile musculature. Surgical excision was performed through a vertical glans incision.

Intraoperatively, an encapsulated mass was carefully dissected from surrounding adhesions and excised completely. Frozen section analysis showed no malignant cells. Histopathological examination revealed a spindle cell neoplasm with Antoni A and Antoni B areas, nuclear palisading, myxoid stromal change, collagenization, and sclerosis, with no mitotic activity or atypia. Immunohistochemistry demonstrated strong, diffuse S-100 positivity, consistent with a conventional schwannoma.

The patient was reviewed clinically at three months, six months, and annually thereafter. At the four-year follow-up, he remained asymptomatic, with preserved erectile and urinary function and no evidence of recurrence or penile deformity.

Penile schwannomas are rare benign tumors with an excellent prognosis after complete excision. Preoperative diagnosis is often limited by nonspecific clinical and imaging findings; however, MRI contributes significantly by delineating lesion characteristics and surgical planes. Histopathology with immunohistochemistry remains essential for definitive diagnosis. Recurrence, though uncommon, has been reported; hence, long-term follow-up is advisable. Even in rare locations such as the glans, schwannoma should be considered in the differential diagnosis of penile masses.

## Introduction

The vast majority of peripheral nervous system cancers originate from Schwann cells, rather than from the nerve cells themselves. These tumors are commonly termed schwannomas or neurilemmomas and are typically benign. They may occur as isolated lesions or as part of underlying clinical syndromes such as neurofibromatosis [1]. Schwannomas are frequently associated with alterations in the neurofibromatosis type 2 (NF2) gene located on chromosome 22, which encodes the protein merlin. Neurilemmomas (NL) may occur sporadically or in association with conditions such as Carney complex, NF2, and schwannomatosis [2-3]. The neck, retroperitoneum, and mediastinum are the most commonly involved sites, while lesions in the extremities typically affect the flexor surfaces. Although the majority of schwannomas are benign, malignant transformation has been infrequently reported [1]. Although the external genitalia have quite rich innervation, the incidence of benign or malignant subcutaneous penile lesions is rare. Penile schwannomas most commonly present in patients with a mean age of 39.2 years, with a median lesion size of approximately 2 cm (range 1.07-3.25 cm) [4].

In 1970, the first three cases of penile schwannoma were reported by Dehner and Smith [5]. To date, only 47 cases of neurilemmoma have been reported in the English literature [6-7]. Penile schwannomas are usually painless, asymptomatic, sporadic, and slow-growing tumors. The dorsal penile shaft within Buck's fascia is the most commonly affected site. While schwannomas in other locations usually present as solitary lesions, approximately 30% of penile schwannomas may be multifocal [8]. Lesions involving the glans or prepuce may be associated with erectile dysfunction, painful intercourse, or penile curvature. Variability in tumor vascularity has been described, with Reynolds et al. reporting increased vascularity and prominent arterial flow in approximately half of cases [9].

The differential diagnosis of any superficial tumor affecting the penis includes atheroma, lipoma, fibroma, fibrosis from autoinjection, Peyronie's disease, and schwannoma. Penile schwannoma can be differentiated from atheroma and lipoma because the latter are more superficial and softer. Schwannoma is differentiated from injection-related fibrosis or Peyronie's disease on the basis of its characteristic site being the dorsal penile shaft [10].

Diagnosis is established through excisional biopsy and supported by imaging modalities such as ultrasonography and magnetic resonance imaging [2,7]. The histopathological appearance of penile schwannoma is characterized by the presence of spindle-shaped cells with a mixture of Antoni A and Antoni B patterns, which often show palisading [1]. Immunohistochemical studies are essential for establishing the diagnosis, using the anti-S-100 protein antibody [1,2]. 

Surgical excision with negative margins remains the treatment of choice. As schwannomas arise from Schwann cells rather than nerve axons, they are typically well-encapsulated, allowing careful dissection while preserving neural structures [2]. However, surgical management in the penile and glans region requires particular caution due to the risk of postoperative curvature, disfigurement, and erectile dysfunction [2,11]. This case report highlights the successful management of a rare schwannoma of the glans penis, addressing both diagnostic and surgical challenges associated with this uncommon location.

## Case presentation

A sexually active 33-year-old male patient reported with a painless, palpable, slow-growing penile mass on the dorsal surface of the glans penis for two months. The mass was first noticed approximately one year prior to presentation, initially measuring less than 1 cm, and had gradually progressed to its current size of approximately 2 cm, becoming more apparent to the patient over the preceding two months. The patient denied any history of trauma to the penile or scrotal region, recent weight loss, or neurological symptoms suggestive of spinal cord involvement. Family history of any genetic nerve sheath tumor was also not reported. On examination, the lesion showed no signs of inflammation or associated urinary symptoms. Local examination revealed a solitary, non-tender, nodular mass measuring approximately 2 × 2 cm, soft to firm in consistency, and moderately mobile on bidigital palpation. The mass was located on the dorsal penile shaft within Buck’s fascia, near the prepuce. There was no evidence of regional inguinal lymphadenopathy. The overlying skin was intact, with no café-au-lait macules noted elsewhere on the body. The lesion was not adherent to adjacent tissues. General physical examination showed that all the vital signs were within the normal limits. Blood investigations and urine biochemical findings were within the normal limits.

**Figure 1:**
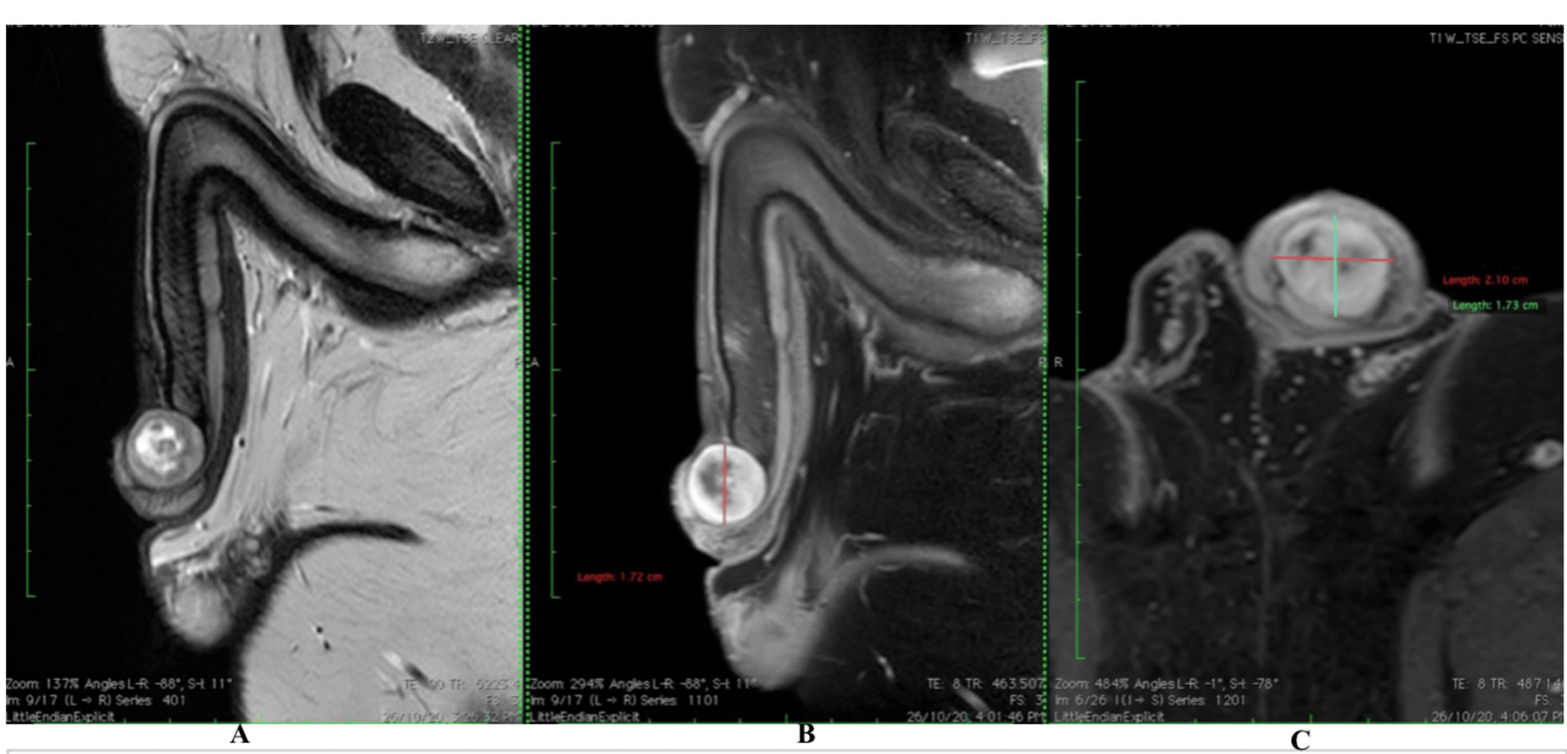
MRI of the penis showing a well-circumscribed glans mass (A) Sagittal T2-weighted image showing a well-circumscribed ovoid mass within the glans with heterogeneous signal intensity and internal hyperintense cystic/necrotic areas. (B) Sagittal post-contrast T1-weighted fat-suppressed image demonstrating avid enhancement of the lesion. (C) Axial post-contrast T1-weighted fat-suppressed image showing the enhancing mass abutting but not invading the corpora cavernosa, corpus spongiosum, and penile urethra.

Preoperative diagnosis was aided by ultrasonography and contrast-enhanced multiplanar MRI of the penis. Penile ultrasonography suggested a hypoechoic mass extending from the base to the dorsal side of the glans penis, measuring around 2.5 × 2.0 cm. The mass showed a clear boundary distinguishing it from the penile corpus cavernosum, and with internal linear echogenic septa. Plain and contrast-enhanced multiplanar MRI of the penis revealed a well-circumscribed, rounded soft-tissue mass along the dorsal aspect of the glans penis, measuring approximately 2.1 × 1.7 × 1.7 cm in maximum axial and longitudinal dimensions. The lesion was iso-intense on T1-weighted images and showed heterogeneous intermediate signal on T2-weighted sequences with internal T2 hyper-intense, non-enhancing cystic/necrotic areas. A thin T2 hypo-intense rim was also noted. Following contrast administration, the mass demonstrated avid heterogeneous enhancement without washout. The lesion was seen abutting and compressing the terminal portions of the corpora cavernosa, corpus spongiosum, and penile urethra; however, no definite invasion of these structures was identified. The overlying skin appeared uninvolved, and the remainder of the penis was normal. No significant abnormality was detected elsewhere in the pelvis, and no regional lymphadenopathy or distant pelvic lesion was noted. The overall features were most consistent with a well-circumscribed enhancing penile neoplasm, with histopathological correlation required for confirmation, as demonstrated on MRI(Figure 1A-1C).

**Figure 2:**
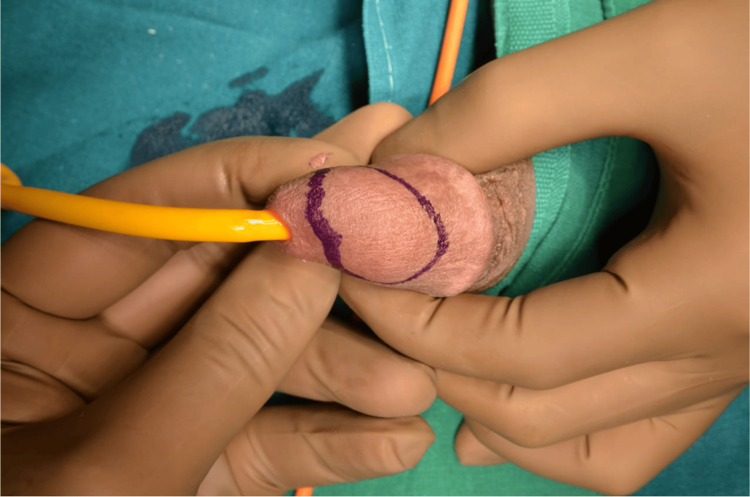
Intraoperative photograph showing the glans penis with a well-demarcated swelling Image obtained prior to excision, demonstrating lesion location and operative orientation. A Foley catheter is in situ to assist with urethral identification.

Based upon the thorough case history, clinical examination, and radiological investigations, the diagnosis of a probable benign penile mass was made. The differential diagnosis of lesions such as penile lipoma, leiomyomas, schwannoma, atheroma, fibroma, and their malignant counterparts was considered. Due to the size of the tumor and its increasing trend, a surgical removal was planned under general anesthesia. After the patient had been placed in a supine position, the usual preparations were completed, and a Foley catheter was inserted for urethral identification. The well-demarcated glans swelling corresponding to the lesion was outlined with a surgical marker, as demonstrated intraoperatively (Figure 2). A vertical incision was made over the skin of the glans directly above the palpable mass. The alveolar tissues were carefully dissected and retracted to expose Buck’s fascia. An encapsulated, well-defined, rounded mass was identified within the glans. The lesion was meticulously separated from surrounding adhesions and excised in toto. An intraoperative frozen section was performed, which revealed no evidence of malignant cells. Hemostasis was achieved. The incision was closed in layers, with the skin approximated using 4-0 Monocryl in a subcuticular manner.

**Figure 3:**
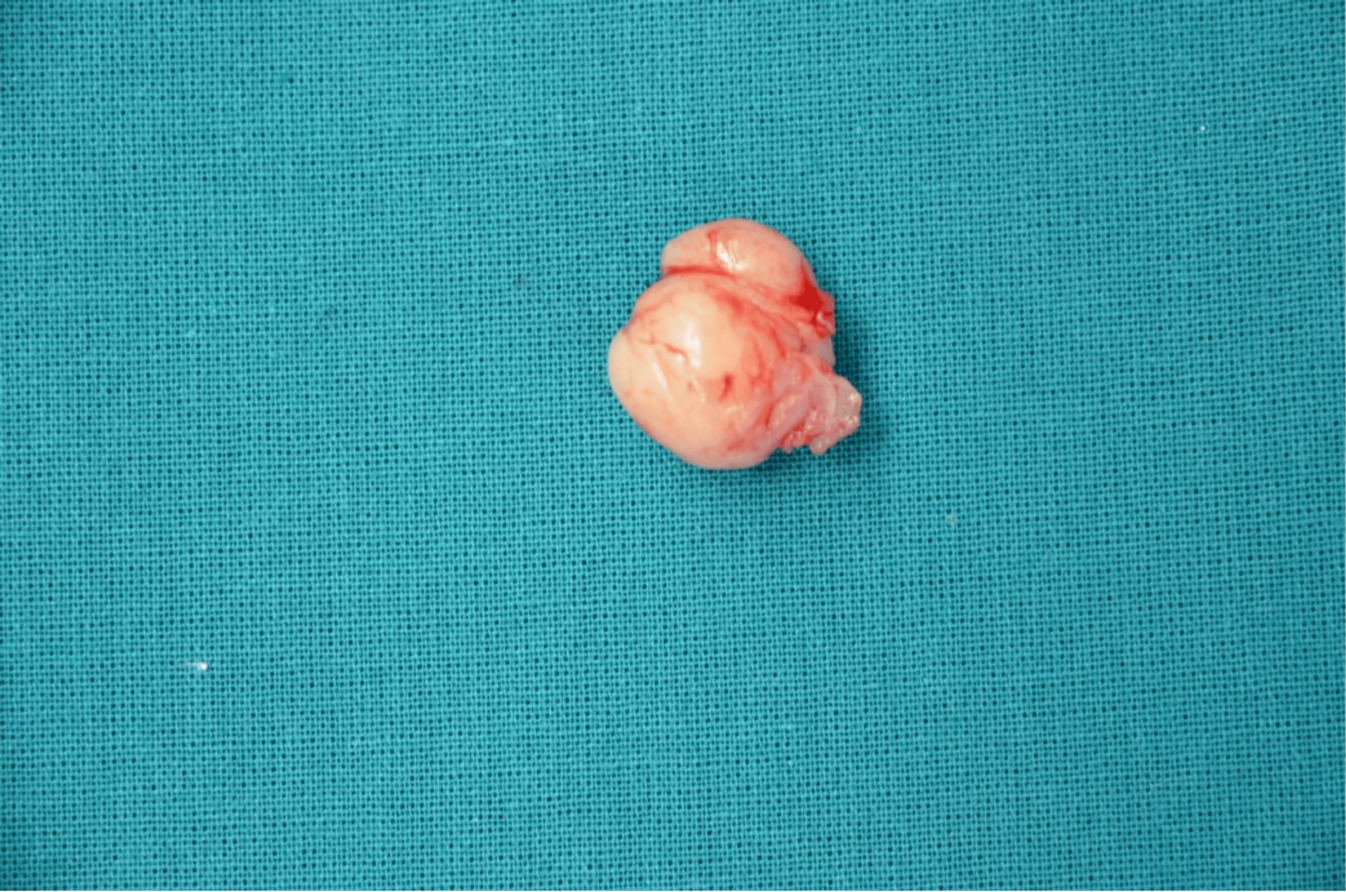
Excised mass specimen Excised specimen from the glans penis showing a well-circumscribed, encapsulated, tan-white soft-tissue mass measuring 2.6 × 1.7 cm.

**Figure 4:**
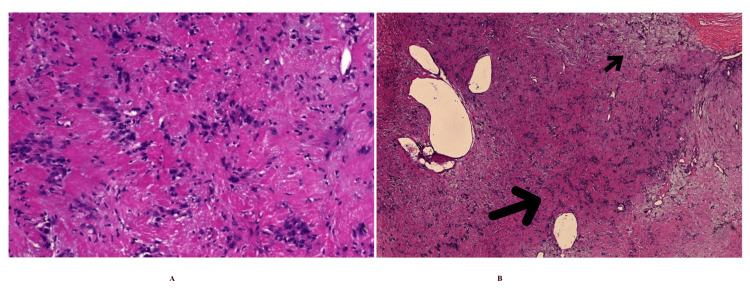
Histopathological examination (hematoxylin and eosin stained sections) of the excised lesion (A) High-power view (×200) of a hypercellular Antoni A area showing intersecting fascicles of spindle cells with nuclear palisading and the formation of Verocay bodies, characterized by central eosinophilic acellular zones. (B) Low-power view demonstrating alternating Antoni A and Antoni B areas. The Antoni A areas (large arrow) are hypercellular and composed of densely packed spindle cells with nuclear palisading, while the Antoni B areas (small arrow) are hypocellular, loosely textured, and myxoid, with focal collagenization. These features are consistent with schwannoma (neurilemmoma).

The excised mass measured 2.6 × 1.7 cm on gross examination and was submitted for histopathological evaluation, as shown in Figure 3. Microscopy revealed a well-circumscribed spindle cell neoplasm exhibiting nuclear palisading with alternating hypercellular Antoni A and hypocellular Antoni B areas, along with myxoid stromal changes and focal collagenization and sclerosis. On immunohistochemistry, the tumor cells demonstrated strong and diffuse positivity for S-100 protein, supporting the diagnosis of schwannoma (neurilemmoma), as illustrated in Figure 4A-4B.

Following surgical excision, the patient was reviewed at three months, six months, and subsequently on a yearly basis with clinical examination. Early follow-up demonstrated satisfactory wound healing, normal erectile function, and uneventful micturition, as assessed by patient-reported outcomes during clinical visits. On continued surveillance for four years, the patient remained asymptomatic, with no evidence of erectile dysfunction, penile curvature, or local recurrence.

## Discussion

Benign penile neoplasms are rare, and penile schwannomas are exceptionally uncommon. This case highlights the diagnostic and surgical considerations in managing a schwannoma of the glans penis, with a favourable long-term outcome. Our patient, a 33-year-old man, presented with a painless, slow-growing penile mass, a clinical presentation consistent with previously reported cases of penile schwannoma (Table 1). While most cases are described as solitary lesions with an indolent course, Gkekas et al. reported a rare plexiform variant arising from the dorsal penile shaft with two distinct nodules at presentation, highlighting the histological and anatomical heterogeneity of these tumors [11]. In contrast, Kumar et al. described a much younger presentation, with diagnosis in a 16-year-old boy [12], while Mondal et al. reported a 62-year-old male with a painless, slowly enlarging dorsal penile schwannoma without associated symptoms [8].

**Table 1:** Comparison of reported penile schwannomas in the literature and the present case The above table illustrates reported cases of penile schwannoma in recent studies and compares them with the present case. These tumors demonstrate a wide age range at presentation and typically follow an indolent clinical course, most commonly manifesting as painless, slowly enlarging penile masses, with an average tumor size of approximately 2–3 cm. Most reported cases have been managed with complete surgical excision. * Systematic literature review.

Recent Studies	Age (years)	Location	Size (cm)	Symptoms	Treatment	Outcome
Nguyen et al. (2016)* [2]	39.2 (mean)	Shaft / glans	~2.0 (average)	Usually painless mass	Surgical excision + adjuvant therapy (malignant cases)	Complete remission
Mondal et al. (2017) [8]	62	Dorsal shaft near prepuce	3	Painless mass	Surgical excision + circumcision	No recurrence
Kumar et al. (2017) [12]	16	Penile (presenting as scrotal mass)	6 x 7	Painless, slowly growing mass	Surgical excision	No recurrence
Gkekas et al. (2019) [11]	39	Dorsal shaft	2 × 1; 0.5	Mild pain, curvature	Surgical excision	No recurrence
Kim et al. (2021) [4]	38	Penoscrotal junction (penile root)	2.1	Painless palpable mass, increased in size during erection	Surgical excision	No recurrence
Present case	33	Glans penis	2.0	Painless mass	Surgical excision	No recurrence

The role of imaging in the preoperative evaluation of penile schwannomas remains adjunctive. Jung et al. reported that ultrasonography may be limited in characterizing schwannomas, as typical features such as a hyperechoic center with a hypoechoic periphery may not always be appreciable [7]. In contrast, MRI provides superior soft-tissue characterisation. Schwannomas typically demonstrate T1 iso to hypo-intensity, T2 hyper-intensity, and post-contrast enhancement, with heterogeneity reflecting myxoid or cystic change [7,13]. In the present case, contrast-enhanced multiplanar MRI demonstrated a well-circumscribed, encapsulated appearing lesion with a thin peripheral low-signal rim suggestive of a capsule, heterogeneous enhancement, and internal cystic changes. The absence of infiltrative margins, preservation of adjacent structures, and small lesion size favored a benign tumor over malignant transformation, which is more commonly associated with ill-defined borders and larger size. These imaging features, in conjunction with clinical findings, supported the decision for complete surgical excision.

Histopathological examination remains the gold standard for the diagnosis of schwannoma. Schwannomas are broadly classified into melanotic, plexiform, and cellular subtypes, each with distinct biological behaviour [14]. In the present case, histology demonstrated a well-circumscribed spindle cell neoplasm with a biphasic pattern comprising hypercellular Antoni A areas showing nuclear palisading and Verocay body formation, and hypocellular Antoni B areas with myxoid stroma and focal collagenization. No significant cytologic atypia or mitotic activity was identified. Immunohistochemistry showed strong and diffuse S-100 protein positivity. These histomorphological and immunophenotypic features closely mirror those described in previously reported penile schwannomas, which consistently exhibit classic Antoni A/B architecture, low proliferative activity, and benign clinical behavior, thereby supporting the diagnosis of a conventional schwannoma [2,14].

Although several benign and malignant penile lesions were considered, the combined clinicoradiologic and histopathological findings favored a diagnosis of schwannoma. Leiomyoma and leiomyosarcoma were considered unlikely due to the absence of smooth muscle differentiation and the presence of strong, diffuse S-100 protein positivity. Vascular tumors were excluded based on the lack of prominent vascular channels on imaging and histology. Peyronie’s disease and injection-related fibrosis typically arise from the tunica albuginea and present as plaque-like lesions rather than a discrete, encapsulated mass within Buck’s fascia. Neurofibroma was also considered less likely given the presence of a well-defined capsule and characteristic Antoni A and Antoni B architecture, which are not typical of neurofibromas.

Surgical excision remains the treatment of choice for benign penile schwannomas, as these tumors are usually well-encapsulated and separable from adjacent structures. In the present case, complete excision was achieved with preservation of surrounding tissues, and intraoperative frozen section confirmed the absence of malignant features. Malignant peripheral nerve sheath tumors, although exceedingly rare in the penis, should be considered in cases of rapid growth, pain, or infiltrative features and may require multimodal management including surgery with adjuvant therapy [15].

At the four-year follow-up, the patient remained asymptomatic with preserved erectile and urinary function and no evidence of penile curvature or local recurrence, reflecting the favorable prognosis of conventional penile schwannomas following complete surgical excision.

## Conclusions

Schwannoma (neurilemmoma) of the penis is an exceptionally rare benign tumor, with occurrence at the glans being even rarer. Preoperative diagnosis is often difficult due to nonspecific presentation; however, contrast-enhanced MRI may be a preferred imaging modality for delineating lesion characteristics and guiding surgical planning. Histopathology with immunohistochemistry remains the gold standard for confirmation. Complete surgical excision is the treatment of choice, and long-term follow-up is recommended, as recurrence, though uncommon in benign nerve sheath tumors, has been reported.
